# Spontaneous Resolution of Symptomatic Hepatic Sarcoidosis

**DOI:** 10.1155/2018/1535049

**Published:** 2018-08-01

**Authors:** Viva Nguyen, Henry N. Ngo, Hamza H. Awad

**Affiliations:** ^1^Mercer University School of Medicine, Columbus, GA, USA; ^2^Department of Internal Medicine, St. Francis Columbus Clinic, Columbus, GA, USA; ^3^Department of Internal Medicine, Mercer University School of Medicine, Columbus, GA, USA; ^4^Department of Community Medicine, Department of Internal Medicine, Mercer University School of Medicine, Macon, GA, USA

## Abstract

Sarcoidosis is an inflammatory process of unknown etiology, characterized by noncaseating granulomas. Isolated extrapulmonary disease is rare. We present a case of a 60-year-old woman with chronically elevated alkaline phosphatase. Upon obtaining a liver biopsy, granulomatous hepatitis was observed, suggestive of sarcoidosis. No particular treatment was initiated, and 3 years following the onset of elevated alkaline phosphatase, her levels decreased spontaneously.

## 1. Introduction

Sarcoidosis is an inflammatory process of unknown etiology, characterized by noncaseating granulomas. The disease can affect multiple organs of the body, including the liver. Hepatic involvement is common with 50% to 60% of patients having granulomas on liver biopsy, but the majority of patients are asymptomatic [1]. Despite the possible presence of granulomas on biopsy, abnormal liver enzymes, or radiological imaging indicative of disease, most patients do not exhibit any signs or symptoms [2]. In the United States, hepatic sarcoidosis is approximately twice as common among African-Americans compared to Caucasians [3]. Health professionals need to be aware of the possible diagnosis of isolated granulomatous hepatitis due to sarcoidosis. Hepatic sarcoidosis can be resolved without treatment, unlike other organs, such as the lungs. The aim of this case report is to demonstrate that liver involvement in sarcoidosis can be symptomatic. In spite of the abdominal pain and weight loss exhibited by this patient in this case, spontaneous resolution of liver hepatitis required no treatment.

## 2. Case Report

A 60-year-old African American female was following up for her chronically elevated alkaline phosphatase levels. She had a history of hypertension, hyperlipidemia, type 2 diabetes mellitus, allergic rhinitis, and chronic lower back pain. Patient has a family history of arthritis, cardiovascular disease, and diabetes mellitus; she denies ever using alcohol or tobacco.

With the onset of elevated alkaline phosphatase level and vague abdominal pain in 2013, an abdominal ultrasound performed in December showed hepatic steatosis. Viral serologies for hepatitis during 2013 were negative, as a gastrointestinal consult was required to determine the need for a liver biopsy. A liver biopsy was subsequently performed, which showed focal mixed micro and macrovesicular steatosis. Portal tracts showed minimal focal chronic inflammation, no significant fibrosis, and no iron deposition.

**Figure 1:**
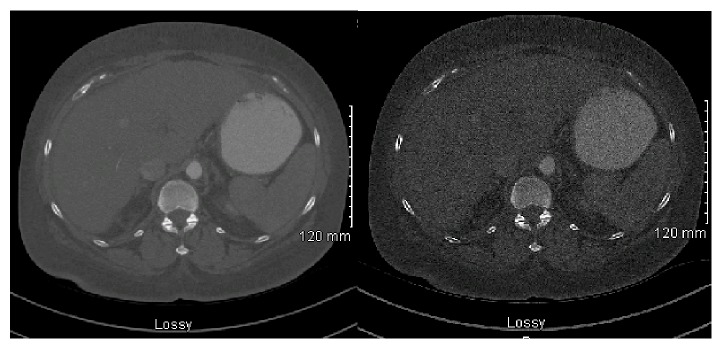
Abdominal Computed Tomography (CT), 2015.

**Figure 2:**
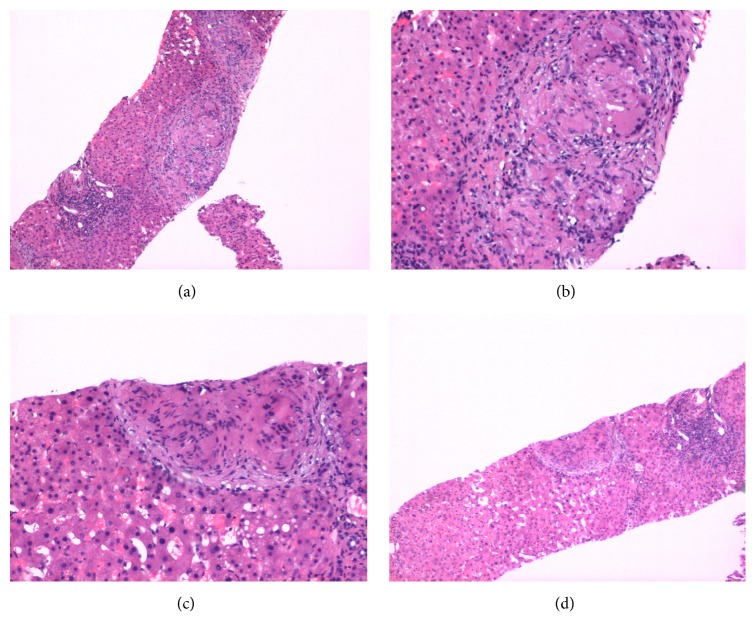
Liver biopsy, 2015. (a) Liver, H+E, 40x, low power view showing liver parenchyma with focal replacement by noncaseating granulomas. (b) Liver, H+E, 200X, high power view demonstrating noncaseating granulomas and giant cells. (c) Liver, H+E, 200X, high power view demonstrating noncaseating granulomas and giant cell. (d) Liver, H+E, 40x, low power view demonstrating noncaseating granuloma (center).

The vague abdominal pain that she was experiencing waxed and waned for two years. Additionally, the patient experienced some vague chest pain and dyspnea that prompted an echocardiogram in February of 2015, which demonstrated a left ventricle ejection fraction of 44%. Consequently, a left heart catheterization in the following month showed no significant coronary disease with a dilated left ventricle with an ejection fraction of 50%. A 2-year follow-up in July of 2015 showed suspicious cirrhosis by Computed Tomography (CT) scan (Figure 1), possibly due to granulomatous changes and chronic inflammation. A CT scan was determined to be necessary for our patient because of rising alkaline phosphatase without other explainable etiologies, in addition to the patient's appetite suppression and vague abdominal pains. Patient's weight during this time was 207 lbs (93.89 kg) and was advised to diet and exercise. After 4 months of continuous symptoms, especially with abdominal pain, a laparoscopic cholecystectomy was performed with a liver biopsy that showed subsequent granulomatous changes in September of 2015. The liver biopsy showed coalescing periportal nonnecrotizing epithelioid granulomas with associated multinucleated giant cells and chronic inflammation. The chronic and patchy inflammation is representative of the granulomatous hepatitis, despite not having elevated transaminases. The granulomatous changes suggested possible sarcoidosis (Figure 2). The liver biopsy was not histologically suggestive of nonalcoholic steatohepatitis, with no steatosis noted. Chest X-ray at that time showed no significant findings.

Inflammatory bowel disease (IBD) was not explored in the patient because she never had clinical signs on past or present examinations, denying any symptoms of IBD including alternating bowel habits, predominant constipation, or diarrhea. Laboratory studies 2 years and 6 months since the onset, at the end of 2015 showed negative antinuclear antibodies (ANA), negative rheumatoid arthritis factor, negative cyclic citrullinated peptide (CCP) antibodies (IgG/IgA), elevated C-reactive protein of 8.9 mg/L, normal complement C3/C4, elevated B-type natriuretic peptide 688.6 pg/mL, and negative mitochondrial (M2) antibody. Subsequent office visits and additional laboratory results are shown in Table 1.

Additionally, patient experienced peak weight loss with a weight of 154 lbs. (69.85 kg) in 2016. During this time period, an echocardiogram showed a decreased ejection fraction of 26%. This result and a history of having ventricular tachycardia resulted in the patient having an automated implantable cardioverter-defibrillator (ICD) placed.

Urinalysis in June 2017 showed RBC 0-5/hpf, WBC 0-5/hpf, bacteria 2+, and moderate calcium oxalate crystals. Additionally, patient's weight increased in 2017 to 190 lbs. (86.18 kg).

## 3. Discussion

Sarcoidosis can affect many different organ systems, leading to long term or severe disease if left untreated with up to 7% risk of death [4]. The spectrum of liver disease in sarcoidosis is broad. The majority have hepatic granulomas on liver biopsy, while some have elevated serum liver enzymes and hepatomegaly. Serum aminotransferases can rise up to 70% in those with hepatic sarcoidosis, but the degree of increase in serum alkaline phosphatase is greater, relative to the liver transaminases [5]. Hepatomegaly is seen in 5% to 15% and cirrhosis develops in 6% of patients with hepatic sarcoidosis [1, 5]. However, the evidence of organ dysfunction is rare. Therefore, the diagnosis of sarcoidosis with liver involvement can be difficult with asymptomatic patients or those with mild symptoms.

Elevations in alkaline phosphatase, as seen in our patient, and/or gamma glutamyltranspeptidase are indications of liver and biliary involvement. Alkaline phosphatase can rise up to 10-fold the upper limit of normal. While elevated alkaline phosphatase is specific to any disease or condition, the patient's medication was excluded as a cause, as alkaline phosphatase remained high despite adjustment and elimination of the medications. With our patient, the alarming factor was the consistently elevated alkaline phosphatase with weight loss. Aminotransferases elevations tend to be milder and less frequent compared to the alkaline phosphatase [1]. With our patient, after her laparoscopic cholecystectomy, one would expect her liver enzymes to decrease. However, her alkaline phosphatase levels remained highly elevated. The ongoing weight loss, fortunately, seemed to have helped her with her uncontrolled diabetes, as indicated by the decline in hemoglobin A1c from 13.0% to 9.7%. Angiotensin converting enzyme (ACE) levels were not obtained because of the insensitivity of the test and was determined to be unhelpful in the diagnosis of sarcoidosis.

Radiological studies, such as ultrasonography or Computed Tomography, may show hepatomegaly or hypoattenuated nodules in the liver. These nodules may be confused with liver metastasis or other granulomatous diseases [6]. Our patient's CT in 2015 initially showed possible cirrhosis with granulomatous changes in a span of 3 months since the onset of major symptoms. No hepatomegaly was noted during that time.

Liver biopsy should be considered if the diagnosis is uncertain, as was done with our patient. Moderate to severe liver-test abnormalities indicate a need for liver biopsy [7, 8]. Histopathological examination is a definitive tool; however, liver biopsy is not histologically suggestive of nonalcoholic steatohepatitis. In sarcoidosis, noncaseating and epithelioid granulomas are seen in the periportal and the portal regions of the liver. Sarcoid epithelioid granulomas are characterized by macrophages that form giant cells surrounded by fibrin rings. In some cases, granulomas may be due to hepatitis C or prior exposure to interferon therapy for hepatitis C. Other causes of granulomatous lesions in the liver, such as tuberculosis, fungal infections, primary biliary cholangitis, Hodgkin disease, and drug toxicity, should be considered before making a diagnosis of sarcoidosis.

Therapeutic regimen for hepatic sarcoidosis remains undefined. Observation alone is sufficient for patients with asymptomatic liver disease or isolated mild elevations of serum liver enzymes. Hepatomegaly alone is not an indication for treatment. In some asymptomatic patients, abnormal serum liver tests can resolve spontaneously or remain stable for many years. With the weight loss and decrease in appetite, the concern became whether or not to initiate any treatment. What if the patient was particularly thin and experienced these weight losses? Would that have changed the management? Our patient demonstrated spontaneous improvement in her alkaline phosphatase levels, along with her aminotransferases. The time frame of approximately 1.5 years demonstrated the gradual decline in the alkaline phosphatase levels and improvement of symptoms. One could predict that her alkaline phosphatase levels should continue to improve, despite not being on any particular treatment for the sarcoidosis.

Death of patients with sarcoidosis usually occurs from severe pulmonary, cardiac, and central nervous system disease, rather than hepatic involvement [9]. Chronic hepatic sarcoidosis may lead to portal hypertension and cirrhosis. When managing treatment of sarcoidosis, consultations with specialists are important due to the multiorgan effect of the disease, especially with the pulmonary and central nervous system. The need to start systemic glucocorticoids was considered, but after weighing the risks and benefits, the patient opted to observe and wait with a watchful eye on possible complications.

The plan for our patient will be to continue monitoring her liver functions, as well as monitoring her pulmonary, cardiac, and central nervous systems with the appropriate specialist, for any developments. Our patient had systolic heart failure and tachyarrhythmia that required an ICD. Cardiac sarcoidosis can manifest as heart blocks, arrhythmias, and heart failure. While findings of the echocardiogram did not suggest cardiac involvement, further cardiac work-up in the form of cardiac magnetic resonance (CMR) imaging, nuclear imaging, cardiac metaiodobenzylguanidine (MIBG) imaging, or endomyocardial biopsy might be warranted for confirmation of cardiac sarcoidosis, bearing in mind the limitations of these modalities based on their sensitivity, specificity, availability, and local expertise.

## Figures and Tables

**Table 1:** Laboratory values at subsequent follow-ups.

Laboratory	November 2016	March 2017	June 2017	Normal Value
WBC	5.8	5.5	6.3	3.0-11.0 K/*μ*L
RBC	5.30	5.30	4.61	4.20-5.40 M/*μ*L
Glucose:	366	402	245	65-99 mg/dL
Calcium:	9.9	9.9	9.1	8.6-10.3 mg/dL
Total Protein:	8.6	8.2	6.8	6.0-8.3 g/dL
Albumin:	3.4	4.0	3.6	3.5-5.7 g/dL
Total bilirubin:	1.8	1.0	0.5	0.3-1.0 mg/dL
Alkaline Phosphatase:	510	338	226	34-104 IU/L
AST:	42	34	20	13-39 IU/L
ALT:	32	30	16	7-52 IU/L
Magnesium:	1.4			1.9-2.7 mg/dL
Hemoglobin A1c:		13.0%	9.7%	4.0-6.0%
