# Corrigendum to “Spontaneous Resolution of Symptomatic Hepatic Sarcoidosis”

**DOI:** 10.1155/2018/8603896

**Published:** 2018-11-06

**Authors:** Viva Nguyen, Henry Ngo, Hamza H. Awad

**Affiliations:** ^1^Mercer University School of Medicine, Columbus, GA, USA; ^2^Department of Internal Medicine, St. Francis Columbus Clinic, Columbus, GA, USA; ^3^Department of Internal Medicine, Mercer University School of Medicine, Columbus, GA, USA; ^4^Department of Community Medicine, Department of Internal Medicine, Mercer University School of Medicine, Macon, GA, USA

In the article titled “Spontaneous Resolution of Symptomatic Hepatic Sarcoidosis” [1], the name of the second author was given incorrectly as Henry N. Ngo. The author's name should have been written as Henry Ngo. The revised authors' list is shown above.
